# Analysis of gut microbiota in patients with cerebral autosomal dominant arteriopathy with subcortical infarcts and leukoencephalopathy (CADASIL)

**DOI:** 10.3164/jcbn.19-22

**Published:** 2019-11-01

**Authors:** Jun Matsuura, Ryo Inoue, Tomohisa Takagi, Sayori Wada, Akiko Watanabe, Takashi Koizumi, Mao Mukai, Ikuko Mizuta, Yuji Naito, Toshiki Mizuno

**Affiliations:** 1 Department of Neurology, Graduate School of Medical Science, Kyoto Prefectural University of Medicine, 465 Kajii-cho, Kamigyo-ku, Kyoto 602-8566, Japan; 2 Graduate School of Laboratory of Animal Science, Kyoto Prefectural University, Sakyo-ku, Kyoto 606-8522, Japan; 3 Department of Gastroenterology, Graduate School of Medical Science, Kyoto Prefectural University of Medicine, 465 Kajii-cho, Kamigyo-ku, Kyoto 602-8566, Japan; 4 Graduate School of Life and Environmental Sciences, Kyoto Prefectural University, Sakyo-ku, Kyoto 606-8522, Japan

**Keywords:** CADASIL, gut microbiota, 16S rRNA, ischemic stroke, disease-modifying factor

## Abstract

Cerebral autosomal dominant arteriopathy with subcortical infarcts and leukoencephalopathy (CADASIL) is a major hereditary small vessel disease caused by mutations in *NOTCH3*. The variations in progression and severity among patients suggest that the CADASIL phenotype is modified by some genetic and environmental factors. Recent studies have shown the potential roles of gut microbiota in human diseases. We hypothesized that gut microbiota modifies the disease phenotype. We performed gut microbial meta 16S rRNA analysis of fecal samples from 15 CADASIL patients and 16 controls. The microbial α- and β-diversities and taxonomy were compared between CADASIL patients and controls and between CADASIL patients with and without an ischemic stroke history. No significant difference in α- or β-diversity was observed in either case-control or subgroup comparisons. In the taxonomic microbial analysis, there was a significant increase in abundance of 6 genera and significant decrease in 2 genera in CADASIL patients compared with controls. There was a significant decrease in abundance of 2 genera in CADASIL patients with compared with those without stroke. This is the first study on CADASIL focusing on gut microbiota. Our findings suggest that gut microbiota modifies the onset and progression of CADASIL.

## Introduction

Drowing evidence supports the potential roles of gut microbiota in human health and diseases, including Parkinson’s disease, multiple sclerosis, and atherosclerotic disease.^(1,2)^ It may be difficult to clarify the influence of gut microbiota on sporadic cerebral infarction because stroke patients show different conventional risk factors, including hypertension, smoking, diabetes, hyperlipidemia, and aging. In contrast, patients with cerebral autosomal dominant arteriopathy with subcortical infarcts and leukoencephalopathy (CADASIL), a major hereditary small vessel disease caused by mutations in *NOTCH3*,^(3)^ generally have no common vascular risk factors. Typical clinical characteristics of CADASIL patients are migraines, recurrent ischemic strokes, vascular dementia, and specific white matter lesions with multiple lacunar infarcts.^(4)^ It is well-known that the progression and severity of CADASIL show both inter- and intra-familial variations among patients. Age at onset of the first stroke ranges from 20–70 years old, and moreover, some patients remain asymptomatic even after 70 years old.^(4,5)^ These suggest that the CADASIL phenotype is modified by some genetic or environmental factors in addition to *NOTCH3* mutation. To clarify the contribution of gut microbiota to cerebral infarction, we focused on the profile of fecal microbiota in CADASIL patients.

## Materials and Methods

### Participants

We recruited nineteen Japanese CADASIL patients who visited our outpatient clinic at University Hospital, Kyoto Prefectural University of Medicine from January to November 2017. Eighteen of their family members (15 spouses, 2 siblings, and a child) were recruited as controls. Nutrition survey during the one month before and up to fecal sampling, using the brief-type self-administered diet history questionnaire (BDHQ).^(6)^ Four CADASIL patients and 2 controls were excluded from the analysis because of taking medication that may interfere with gut microbiota (antibiotics: *n* = 1, proton pump inhibitors: *n* = 4, Metformin: *n* = 1). We finally analyzed fecal samples from 15 CADASIL patients and 16 controls. CADASIL patients were divided into two subgroups: those with a history of symptomatic ischemic stroke (*n* = 7) and those without it (*n* = 8). The Ethics Committee of Kyoto Prefectural University of Medicine approved the research protocol (permission No. ERB-C-725-1), and all participants provided written informed consent prior to enrollment.

### Meta16S analysis of gut microbiota

Meta16S analysis of gut microbiota was performed according to Takagi *et al.*^(7)^ In brief, fecal sampling was performed using a sampling kit (Techno Suruga Lab., Shizuoka, Japan). Meta16S analysis of fecal DNA was performed at the Biomedical Center, Takara Bio. PCR for DNA extracted from fecal samples was comprised of two steps. In the first step, PCR was performed to amplify with primers of 341F (5'-TCGTCGGCAGCGTCAGATGTGTATAAGAGACAGCCTACGGGNGGCWGCAG-3') and 806R (5'-GTCTCGTGGGCTC GGAGATGTGTATAAGAGACAGGGACTACHVGGGTWTCTAAT-3') corresponding to the V3–V4 region of the 16S rRNA gene. In the second PCR step, the index sequences for Illumina sequencer were added. Then, the libraries were subjected to the sequencing of paired-end 300 bases on MiSeq (Illumina).

Processing of sequence data, operational taxonomic unit (OTU) definition, and taxonomy assignment were performed using QIIME ver. 1.9, USEARCH ver. 8.0, and UCHIME ver. 4.2.40, respectively. Singletons were removed in the present study. Taxonomy assignment of the resulting OTU was completed using RDP classifier ver. 2.10.2 with the Greengenes database (May 2013).

Microbiota diversity within a sample (α-diversity) was evaluated as the Chao1 index (OTU richness estimation) and Shannon index (OTU evenness estimation) calculated by the R “phyloseq” package. Differences in diversity between samples (β-diversity) was estimated using the UniFrac metric by quantitative (weighted) or qualitative (unweighted) analysis, and the distances between the samples were visualized by principal coordinate analysis (PCoA) plotting, using the software QIIME (ver. 1.9.0).

### Statistical analysis

Data were compared between groups (CADASIL patients vs controls, and CADASIL patients with and without stroke). Nutrition intake profiles were compared by the Mann-Whitney *U* test using SPSS 17.0. The relative abundance of bacterial genera, OTU, and α-diversity were compared with a Wilcoxon rank sum test using JMP14.0.0. Clustering on PCoA plot (β-diversity) and was statistically analyzed using permutational multivariate analysis of variance (PERMANOVA). Values of *p*<0.05 were considered significant.

## Results

### Backgrounds and nutrition survey

There was no significant difference in backgrounds and vascular risk factors between CADASIL patients and controls, or CADASIL subgroups, except for weak difference in gender between CADASIL patients with stroke and those without stroke (*p* = 0.04) (Table 1).

The nutrition survey was performed using a BDHQ.^(6)^ Between CADASIL patients and controls, there was no significant difference in 97 nutrition items. Between CADASIL subgroups, no significant difference was noted except for the daily intake of arachidonic acid (patients with stroke: 22.28 mg/day vs patients without it: 29.39 mg/day; *p* = 0.028).

### Analysis of gut microbiota diversity

**Fig. 1:**
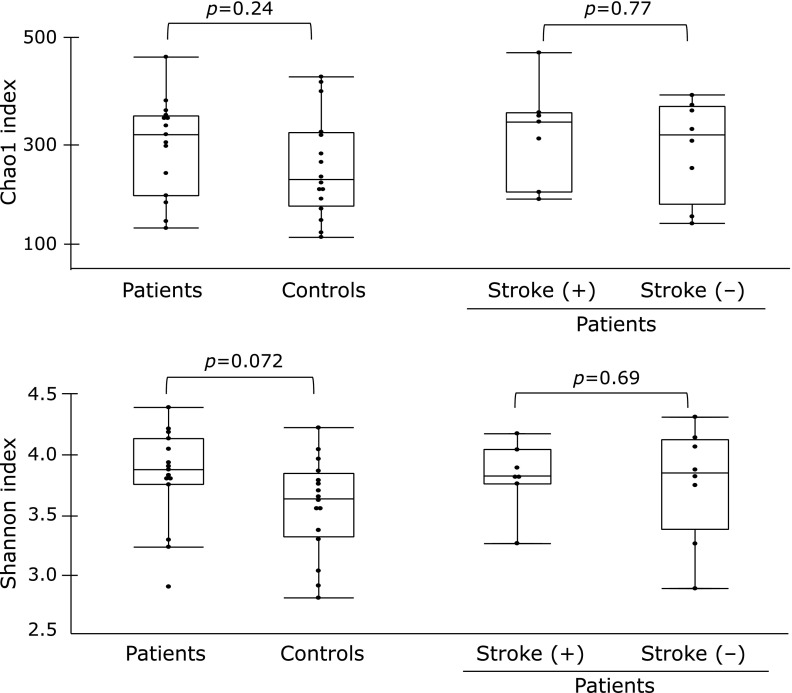
Case-control and subgroup comparison of α-diversity of gut microbiota. Box and whisker plots of the Chao1 index (upper) and Shannon index (lower). These α-diversity indices were compared between CADASIL patients and controls, and between CADASIL patients with previous ischemic stroke and those without it (Wilcoxon rank sum test).

**Fig. 2:**
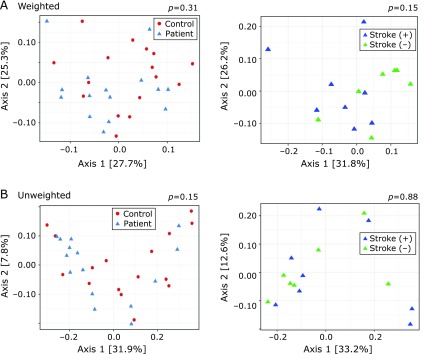
Case-control and subgroup comparisons of β-diversity of gut microbiota. Distribution of β-diversity was visualized by principle coordinate analysis (PCoA) plots from weighted (A) and unweighted (B) UniFrac metrics, and compared between CADASIL patients and controls (left), and between CADASIL patients with previous ischemic stroke and those without it (right) (permutational multivariate analysis of variance).

In total, seven hundred ninety OTUs were detected after the removal of singletons in this study. No significant difference in α-diversity was observed between the CADASIL patients and controls, or between the patients with and without stroke (Fig. 1). As for β-diversity, PCoA plots showed no significant difference in clusters between CADASIL patients and controls, or between CADASIL patients with and without stroke (Fig. 2).

### Taxonomic microbial analysis

The taxonomic change in the microbiota was assessed at genus and OTU levels. In CADASIL patients compared with controls, there was a significant increase in abundance of 6 genera (*Lachnospira*, *Odoribacter*, *Parvimonas*, unclassified genus belonging to *Barnesiellaceae* and *Lachnospiraceae*, unclassified genus belonging to order SHA-98), and a significant decrease in 2 genera (*Megasphaera* and *Acidaminococcus*) (Table 2). Regarding the OTU level, there was a significant increase in abundance of 24 OTUs and significant decrease in 4 OTUs in CADASIL patients compared with controls (Supplemental Table 1*).

In the CADASIL patients with compared with those without stroke, there was a significant decrease in abundance of 2 genera, *Phascolarctobacterium* and *Paraprevotella* (Table 2).

Regarding the OTU level, a significant increase in abundance of 13 OTUs and significant decrease in 3 OTUs were observed in CADASIL patients with compared with those without stroke (Supplemental Table 1*).

## Discussion

To our knowledge, this is the first study addressing the possibility of gut microbiota contributing to the development of CADASIL. The major finding of this study was identification of significant taxonomic differences in gut microbiota between CADASIL patients and controls, and also between patients with and without previous ischemic stroke. These suggest the role of gut microbiota as modifiers of the onset and progression of CADASIL.

Taxonomic analysis showed significant differences in abundances of some bacteria between CADASIL patients and controls. According to previous reports, some of the representative bacteria shown in Table 1 might be associated with stroke onset in CADASIL patients. Firstly, Goodrich *et al.*^(8)^ reported that the abundance of SHA-98, a member of the *Christensenellaceae* consortium, was associated with a gene encoding aldehyde dehydrogenase (ALDH1L1). Interestingly, an SNP in *ALDH1L1* was reported to be associated with ischemic stroke in the Framingham Heart Study.^(9)^ Secondly, *Parvimonas*, a Gram-positive anaerobic coccus, was also of interest according to recent knowledge on the association between periodontal disease and ischemic stroke.^(10)^ Fak *et al.*^(11)^ reported that bacteria from the oral cavity and gut can be recovered from atherosclerotic plaque, and that the abundance of oral *Parvimonas* was positively associated with c-reactive protein (CRP) in atherosclerotic patients. Thirdly, the family *Lachnospiraceae* has been reported to facilitate Treg differentiation and stimulate TGF-β and IL-10 production by immune cells.^(12)^ TGF-β is a key molecule in the pathogenesis of CARASIL, an autosomal recessive small vessel disease caused by *HTRA1* mutations.^(13)^ Dysregulation of TGF-β signaling may also be associated with CADASIL according to a report that latent TGF-β binding protein 1 was sequestrated into CADASIL-related NOTCH3 extracellular domain deposits.^(14)^ Taken together, the increase of *Lachnospiraceae* in CADASIL patients is of interest.

In recent gut microbial taxonomic studies, other bacteria were reported to be associated with ischemic stroke in sporadic patients.^(15)^ In this study, however, we did not replicate any of these findings.

On the other hand, on taxonomic analysis in association with stroke among CADASIL patients, two genera, *Phascolarctobacterium* and *Paraprevotella*, showed a significant difference. Based on our search, however, no previous report addressed the association of these genera with cerebrovascular dysfunction.

A major limitation of our study was the small number of participants, due to the fact that CADASIL is a rare hereditary disease. In this regard, it is of note that Inoue *et al.*^(16)^ reported robust evaluation of gut dysbiosis in type 2 diabetic patients even though the sample size, 12 patients and 10 controls, was as small as our study. By using 16S rRNA metagenomic analysis in combination with functional prediction software Phylogenetic Investigation of Communities by Reconstruction of Unobserved States (PICRUSt) and Kyoto Encyclopedia of Genes and Genomes (KEGG) database, they identified not only composition but also functional profiles of gut microbiota associated with diabetes pathophysiology.^(16)^ Then, we additionally performed similar analysis. Although there was no significant difference between CADASIL patients and normal controls, we found 32 KEGG pathways of which abundances were significantly different between CADASIL patients with and without stroke (Supplemental Fig. 1*). To interpret these findings, further studies including fecal metabolome analysis should be necessary. However, of the 32 pathways, lipid metabolism seems of note, because Žitňanová *et al.*^(17)^ recently reported that subfractions of plasma lipid were significantly altered in acute phase of stroke, another limitation was that we did not adjust background factors to compare gut microbiota between groups. Previous report showed that several changes in gut microbiota were associated with age and sex, by analyzing 277 healthy individuals.^(18)^ Moreover, it cannot be concluded whether taxonomic changes between CADASIL and controls or between CADASIL patients with and without stroke were causes or results of the disease. To address these issues, increasing the number of participants and longitudinal analysis of each patient are necessary.

In conclusion, this is the first study on CADASIL focusing on gut microbiota. Significant differences in taxonomy were noted between CADASIL patients and controls, and between CADASIL patients with and without stroke, suggesting that gut microbiota modifies the onset and progression of CADASIL.

## Author Contributions

TM and YN supervised and designed the study; JM and TM collected feces; JM, AW, TK, MM, IM and TM collected the clinical information; JM, RI, TT, IM, YN and TM analyzed and interpreted the gut microbiota data; JM, SW and TM analyzed and interpreted the nutrition data; JM, RI and SW performed the statistical analysis. All authors drafted and edited the manuscript, and discussed the results and commented on the manuscript.

## Figures and Tables

**Table 1:** Background of the participants

	CADASIL patients (*n* = 15)	Normal controls (*n* = 16)	*p* value	CADASIL patients		*p* value
				Stroke (+) (*n* = 7)	Stroke (–) (*n* = 8)	
Age (range)	56.9 (45–74)	53.7 (28–71)	0.33	57.0 (45–66)	56.9 (47–74)	0.98
BMI	22.9 (18.0–33.0)	22.0 (18.2–29.4)	0.44	23.9 (20.7–30.9)	22.0 (18.0–28.4)	0.3
Male/Female	8 (53%)/7 (47%)	8 (50%)/8 (50%)	1	6 (86%)/1 (14%)	2 (25%)/6 (75%)	0.04
Hypertension	2/15 (13%)	2/16 (13%)	1	2/7 (29%)	0/8 (0%)	0.2
Dyslipidemia	5/15 (33%)	2/16 (13%)	0.22	3/7 (43%)	2/8 (25%)	0.61
Diabetes mellitus	0/15 (0%)	1/16 (6 %)	1	0/7 (0%)	0/8 (0%)	—
Smoking	1/15 (7%)	2/16 (13%)	1	1/7 (14%)	0/8 (0%)	0.47

Mean of age and body mass index (BMI), number of male (%)/female (%), and number of observed/studied (%) are shown. For *p* value calculation, the *t* test (age and BMI), or Fisher’s exact test was employed.

**Table 2:** Genus level taxonomic analysis of gut microbiota

Case-control								
Phylum	Class	Order	Family	Genus	Average ± STDEV (%)			Wilcoxon
					Patients (*n* = 15)	Controls (*n* = 16)		*p* value
Firmicutes	Clostridia	Clostridiales	*Veillonellaceae*	*Megasphaera*	0.000171 ± 0.000513	0.281 ± 0.597		0.001**
Bacteroidetes	Bacteroidia	Bacteroidales	[*Barnesiellaceae*]	Unclassified	0.479 ± 4.69	0.0880 ± 0.148		0.008**
Firmicutes	Clostridia	Clostridiales	*Lachnospiraceae*	Unclassified	0.132 ± 0.142	0.0326 ± 0.0525		0.011*
Firmicutes	Clostridia	Clostridiales	*Lachnospiraceae*	*Lachnospira*	1.44 ± 2.11	0.183 ± 0.348		0.011*
Firmicutes	Clostridia	Clostridiales	*Veillonellaceae*	*Acidaminococcus*	0.0814 ± 0.315	0.405 ± 0.754		0.027*
Firmicutes	Clostridia	SHA-98	Unclassified	Unclassified	0.00459 ± 0.00654	0.000699 ± 0.00176		0.032*
Bacteroidete	Bacteroidia	Bacteroidales	[*Odoribacteraceae*]	*Odoribacter*	0.234 ± 0.224	0.0981 ± 0.0931		0.0326*
Firmicutes	Clostridia	Clostridiales	[*Tissierellaceae*]	*Parvimonas*	0.00287 ± 0.00320	0.00153 ± 0.0038		0.036*

Subgroup								
Phylum	Class	Order	Family	Genus	Average ± STDEV (%)			Wilcoxon
					Stroke (+) (*n* = 7)	Stroke (–) (*n* = 8)		*p* value
Firmicutes	Clostridia	Clostridiales	*Veillonellaceae*	*Phascolarctobacterium*	0.483 ± 0.662	3.48 ± 3.55		0.02*
Bacteroidetes	Bacteroidia	Bacteroidales	[*Paraprevotellaceae*]	*Paraprevotella*	0.0443 ± 0.0837	0.579 ± 0.474		0.028*

The Genera of significant differences (*p*<0.05, Wilcoxon rank test) between CADASIL patients and controls (case-control) and between the CADASIL patients with and without stroke (subgroup) are shown. *p<0.05, **p<0.01.

## References

[1] Yin J, Liao SX, He Y, et al Dysbiosis of gut microbiota with reduced trimethylamine-N-oxide level in patients with large-artery atherosclerotic stroke or transient ischemic attack. J Am Heart Assoc 2015; 4. pii: e002699. 26597155 10.1161/JAHA.115.002699 PMC4845212

[2] Tremlett H, Bauer KC, Appel-Cresswell S, Finlay BB, Waubant E The gut microbiome in human neurological disease: a review. Ann Neurol 2017; 81: 369–382. 28220542 10.1002/ana.24901

[3] Joutel A, Corpechot C, Ducros A, et al Notch3 mutations in CADASIL, a hereditary adult-onset condition causing stroke and dementia. Nature 1996; 383: 707–710. 8878478 10.1038/383707a0

[4] Chabriat H, Joutel A, Dichgans M, Tournier-Lasserve E, Bousser MG Cadasil. Lancet Neurol 2009; 8: 643–653. 19539236 10.1016/S1474-4422(09)70127-9

[5] Mizuta I, Watanabe-Hosomi A, Koizumi T, et al New diagnostic criteria for cerebral autosomal dominant arteriopathy with subcortical infarcts and leukocencephalopathy in Japan. J Neurol Sci 2017; 381: 62–67. 28991717 10.1016/j.jns.2017.08.009

[6] Kobayashi S, Honda S, Murakami K, et al Both comprehensive and brief self-administered diet history questionnaires satisfactorily rank nutrient intakes in Japanese adults. J Epidemiol 2012; 22: 151–159. 22343326 10.2188/jea.JE20110075 PMC3798594

[7] Takagi T, Naito Y, Inoue R, et al The influence of long-term use of proton pump inhibitors on the gut microbiota: an age-sex-matched case-control study. J Clin Biochem Nutr 2018; 62: 100–105. 29371761 10.3164/jcbn.17-78 PMC5773837

[8] Goodrich JK, Davenport ER, Beaumont M, et al Genetic determinants of the gut microbiome in UK twins. Cell Host Microbe 2016; 19: 731–743. 27173935 10.1016/j.chom.2016.04.017 PMC4915943

[9] Williams SR, Yang Q, Chen F, et al Genome-wide meta-analysis of homocysteine and methionine metabolism identifies five one carbon metabolism loci and a novel association of ALDH1L1 with ischemic stroke. PLoS Genet 2014; 10: e1004214. 24651765 10.1371/journal.pgen.1004214 PMC3961178

[10] Wu T, Trevisan M, Genco RJ, Dorn JP, Falkner KL, Sempos CT Periodontal disease and risk of cerebrovascular disease: the first national health and nutrition examination survey and its follow-up study. Arch Intern Med 2000; 160: 2749–2755. 11025784 10.1001/archinte.160.18.2749

[11] Fåk F, Tremaroli V, Bergström G, Bäckhed F Oral microbiota in patients with atherosclerosis. Atherosclerosis 2015; 243: 573–578. 26536303 10.1016/j.atherosclerosis.2015.10.097

[12] Saresella M, Mendozzi L, Rossi V, et al Immunological and clinical effect of diet modulation of the gut microbiome in multiple sclerosis patients: a pilot study. Front Immunol 2017; 8: 1391. 29118761 10.3389/fimmu.2017.01391 PMC5661395

[13] Hara K, Shiga A, Fukutake T, et al Association of HTRA1 mutations and familial ischemic cerebral small-vessel disease. N Eng J Med 2009; 360: 1729–1739. 10.1056/NEJMoa0801560 19387015

[14] Kast J, Hanecker P, Beaufort N, et al Sequestration of latent TGF-β binding protein 1 into CADASIL-related Notch3-ECD deposits. Acta Neuropathol Commun 2014; 2: 96. 25190493 10.1186/s40478-014-0096-8 PMC4243959

[15] Zhao L, Xiong Q, Stary CM, et al Bidirectional gut-brain-microbiota axis as a potential link between inflammatory bowel disease and ischemic stroke. J Neuroinflammation 2018; 15: 339. 30537997 10.1186/s12974-018-1382-3 PMC6290529

[16] Inoue R, Ohue-Kitano R, Tsukahara T, et al Prediction of functional profiles of gut microbiota from 16S rRNA metagenomic data provides a more robust evaluation of gut dysbiosis occurring in Japanese type 2 diabetic patients. J Clin Biochem Nutr 2017; 61: 217–221. 29203964 10.3164/jcbn.17-44 PMC5703784

[17] Žitňanová I, Šiarnik P, Füllöp M, et al Gender differences in LDL- and HDL-cholesterol subfractions in patients after the acute ischemic stroke and their association with oxidative stress markers. J Clin Biochem Nutr 2018; 63: 144–148. 30279626 10.3164/jcbn.17-105 PMC6160728

[18] Takagi T, Naito Y, Inoue R, et al Differences in gut microbiota associated with age, sex, and stool consistency in healthy Japanese subjects. J Gastroenterol 2019; 54: 53–63. 29926167 10.1007/s00535-018-1488-5

