# Supplementary material for: Analysis of gut microbiota in patients with cerebral autosomal dominant arteriopathy with subcortical infarcts and leukoencephalopathy (CADASIL)
Source: J Clin Biochem Nutr. 2019 Nov 1;65(3):240–4. doi: 10.3164/jcbn.19-22 (PMC6877404; doi:10.3164/jcbn.19-22)
Supplement: Supplemental Figure 1 [file jcbn19-22sf01.pdf]

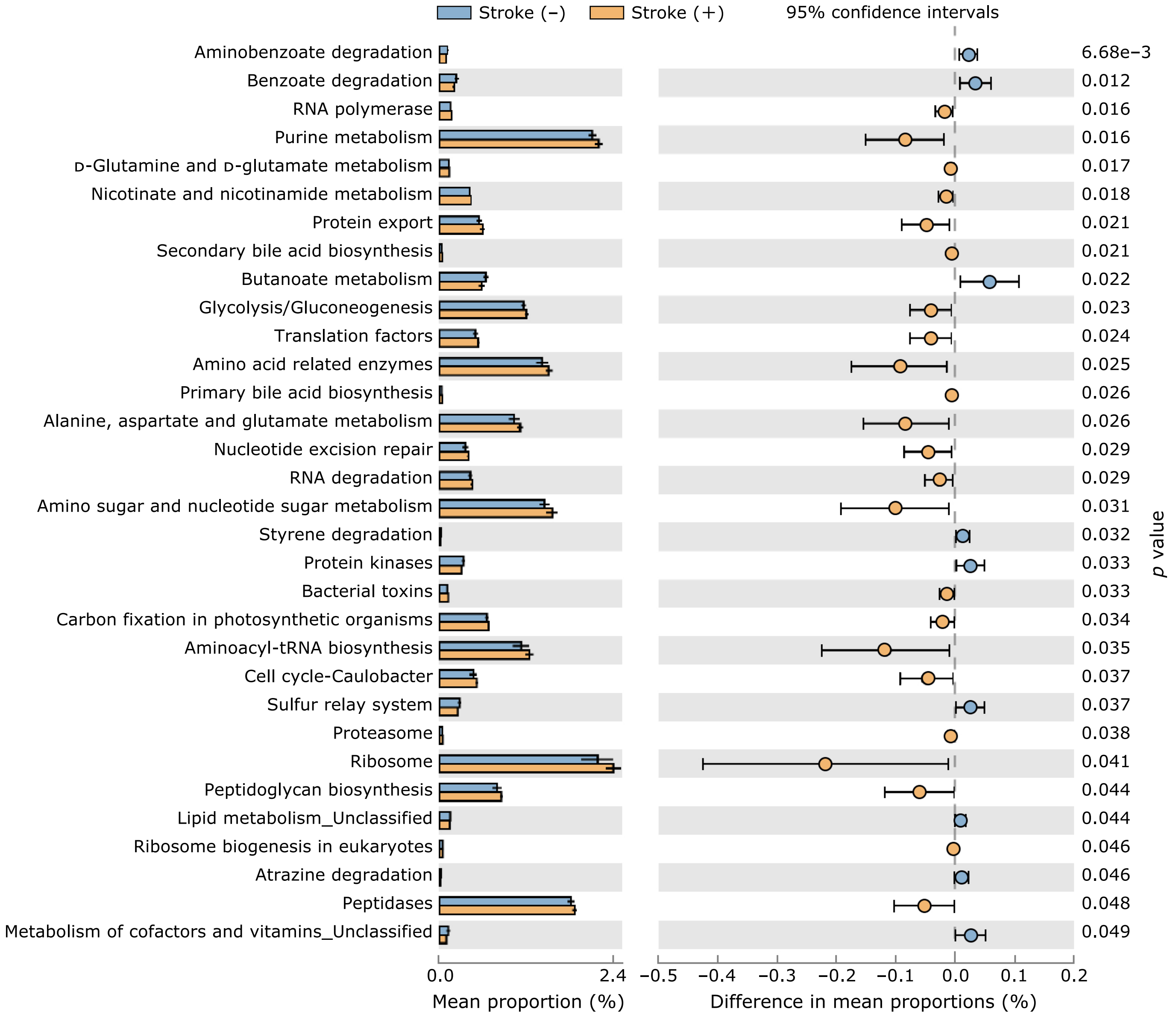

Supplemental Fig. 1. Predicted KEGG pathways in gut microbiota significantly different between CADASIL patients with and without ischemic stroke. Phylogenetic Investigation of Communities by Reconstruction of Unobserved States (PICRUSt) and Kyoto Encyclopedia of Genes and Genomes (KEGG) database were used to predict functional profiles in gut microbiota. 32 pathways of which abundance were significantly different between CADASIL patients with (orange) and without (blue) and *p* values are shown.
