# Supplementary material for: Analysis of gut microbiota in patients with cerebral autosomal dominant arteriopathy with subcortical infarcts and leukoencephalopathy (CADASIL)
Source: J Clin Biochem Nutr. 2019 Nov 1;65(3):240–4. doi: 10.3164/jcbn.19-22 (PMC6877404; doi:10.3164/jcbn.19-22)
Supplement: Supplemental Table 1 [file jcbn19-22st01.pdf]

Supplemental Table 1. OTU (Operational Taxonomic Unit) level taxonomic analysis of gut microbiota

Case-control

OTU	Phylum	Class	Order	Family	Genus	Average ± STDEV (%)		Wilcoxon
						Patient (*n* = 15)	Control (*n* = 16)	*p*
OTU_366	Firmicutes	Clostridia	Clostridiales	*Ruminococcaceae*	*Oscillospira*	0.0189 ± 0.0217	0.00174 ± 0.00429	0.004**
OTU_155	Firmicutes	Clostridia	Clostridiales	*Ruminococcaceae*	*Ruminococcus*	0.0554 ± 0.0769	0.0161 ± 0.0461	0.006**
OTU_352	Firmicutes	Clostridia	Clostridiales	*Ruminococcaceae*	*Ruminococcus*	0.0119 ± 0.0111	0.00227 ± 0.00413	0.007**
OTU_719	Firmicutes	Clostridia	Clostridiales	*Ruminococcaceae*	*Oscillospira*	0.00150 ± 0.00190	0.000159 ± 0.000341	0.007**
OTU_620	Firmicutes	Clostridia	Clostridiales	*Ruminococcaceae*	Unclassified	0.00573 ± 0.0115	0.0000333 ± 0.000129	0.008**
OTU_47	Firmicutes	Clostridia	Clostridiales	*Lachnospiraceae*	*Lachnospira*	1.44 ± 2.04	0.183 ± 0.337	0.011*
OTU_769	Firmicutes	Clostridia	Clostridiales	*Ruminococcaceae*	Unclassified	0.000857 ± 0.00175	0	0.014*
OTU_747	Firmicutes	Clostridia	Clostridiales	*Ruminococcaceae*	*Anaerotruncus*	0.000771 ± 0.00190	0	0.014*
OTU_748	Firmicutes	Clostridia	Clostridiales	*Ruminococcaceae*	Unclassified	0.0135 ± 0.0141	0.00266 ± 0.00420	0.015*
OTU_81	Bacteroidetes	Bacteroidia	Bacteroidales	[*Barnesiellaceae*]	Unclassified	0.434 ± 0.436	0.0708 ± 0.126	0.017*
OTU_214	Firmicutes	Clostridia	Clostridiales	*Ruminococcaceae*	Unclassified	0.0254 ± 0.0368	0.00496 ± 0.00959	0.020*
OTU_113	Actinobacteria	Coriobacteriia	Coriobacteriales	*Coriobacteriaceae*	Unclassified	0	0.189 ± 0.363	0.021*
OTU_100	Bacteroidetes	Bacteroidia	Bacteroidales	[*Odoribacteraceae*]	*Odoribacter*	0.152 ± 0.0869	0.0834 ± 0.0897	0.024*
OTU_536	Firmicutes	Clostridia	Clostridiales	Unclassified	Unclassified	0.00911 ± 0.0189	0.000421 ± 0.00148	0.027*
OTU_49	Firmicutes	Clostridia	Clostridiales	*Veillonellaceae*	*Acidaminococcus*	0.0814 ± 0.304	0.405 ± 0.730	0.027*
OTU_641	Firmicutes	Clostridia	Clostridiales	*Veillonellaceae*	*Megasphaera*	0.000171 ± 0.000495	0.00133 ± 0.00200	0.029*
OTU_677	Firmicutes	Clostridia	Clostridiales	Unclassified	Unclassified	0.00123 ± 0.00270	0	0.030*
OTU_519	Bacteroidetes	Bacteroidia	Bacteroidales	*Bacteroidaceae*	*Bacteroides*	0.0102 ± 0.0383	0.0291 ± 0.0805	0.031*
OTU_587	Firmicutes	Clostridia	Clostridiales	*Dehalobacteriaceae*	*Dehalobacterium*	0.00155 ± 0.00194	0.000246 ± 0.000571	0.032*
OTU_509	Firmicutes	Clostridia	SHA-98	Unclassified	Unclassified	0.00459 ± 0.00632	0.000699 ± 0.00171	0.032*
OTU_487	Firmicutes	Clostridia	Clostridiales	*Christensenellaceae*	Unclassified	0.00175 ± 0.00154	0.000506 ± 0.00134	0.032*
OTU_4	Bacteroidetes	Bacteroidia	Bacteroidales	*Bacteroidaceae*	*Bacteroides*	4.56 ± 3.01	3.14 ± 4.68	0.033*
OTU_38	Firmicutes	Clostridia	Clostridiales	*Lachnospiraceae*	*Coprococcus*	0.609 ± 0.629	0.226 ± 0.309	0.033*
OTU_376	Firmicutes	Clostridia	Clostridiales	[*Tissierellaceae*]	*Parvimonas*	0.00287 ± 0.00310	0.00153 ± 0.00368	0.036*
OTU_427	Firmicutes	Clostridia	Clostridiales	*Lachnospiraceae*	Unclassified	0.0232 ± 0.0353	0.00147 ± 0.00362	0.037*
OTU_292	Firmicutes	Clostridia	Clostridiales	*Christensenellaceae*	Unclassified	0.0124 ± 0.0180	0.00274 ± 0.00526	0.039*
OTU_541	Firmicutes	Clostridia	Clostridiales	*Ruminococcaceae*	*Ruminococcus*	0.0244 ± 0.0200	0.0151 ± 0.0320	0.044*
OTU_720	Firmicutes	Clostridia	Clostridiales	*Ruminococcaceae*	Unclassified	0.00504 ± 0.00927	0.00102 ± 0.00228	0.048*

Subgroup

OTU	Phylum	Class	Order	Family	Genus	Average ± STDEV (%)		Wilcoxon
						Patient (*n* = 7)	Control (*n* = 8)	*p*
OTU_552	Actinobacteria	Actinobacteria	Actinomycetales	*Micrococcaceae*	*Rothia*	0.000182 ± 0.000447	0.00358 ± 0.00426	0.003**
OTU_647	Firmicutes	Clostridia	Clostridiales	*Christensenellaceae*	Unclassified	0.00191 ± 0.0220	0	0.006**
OTU_391	Firmicutes	Clostridia	Clostridiales	*Christensenellaceae*	Unclassified	0.0228 ± 0.0268	0.00167 ± 0.00443	0.013*
OTU_692	Firmicutes	Clostridia	Clostridiales	Unclassified	Unclassified	0.00274 ± 0.00269	0	0.017*
OTU_86	Bacteroidetes	Bacteroidia	Bacteroidales	*Bacteroidaceae*	*Bacteroides*	0.246 ± 0.355	0	0.017*
OTU_805	Firmicutes	Clostridia	Clostridiales	*Ruminococcaceae*	*Anaerotruncus*	0.0185 ± 0.0427	0	0.017*
OTU_766	Actinobacteria	Coriobacteriia	Coriobacteriales	*Coriobacteriaceae*	Unclassified	0.000750 ± 0.000737	0	0.017*
OTU_259	Firmicutes	Clostridia	Clostridiales	*Ruminococcaceae*	*Butyricicoccus*	0.000254 ± 0.000622	0.0201 ± 0.0467	0.024*
OTU_56	Firmicutes	Clostridia	Clostridiales	*Ruminococcaceae*	*Oscillospira*	0.596 ± 0.524	0.150 ± 0.164	0.027*
OTU_33	Firmicutes	Clostridia	Clostridiales	*Lachnospiraceae*	*Blautia*	0.996 ± 0.829	0.876 ± 0.448	0.028*
OTU_799	Tenericutes	Mollicutes	RF39	Unclassified	Unclassified	0.000307 ± 0.00161	0	0.037*
OTU_156	Actinobacteria	Coriobacteriia	Coriobacteriales	*Coriobacteriaceae*	Unclassified	0.0408 ± 0.0528	0	0.047*
OTU_99	Firmicutes	Clostridia	Clostridiales	*Lachnospiraceae*	[*Ruminococcus*]	0.0514 ± 0.102	0.124 ± 0.0342	0.047*
OTU_147	Proteobacteria	Betaproteobacteria	Burkholderiales	*Alcaligenaceae*	*Sutterella*	0.250 ± 0.381	0	0.047*
OTU_89	Firmicutes	Clostridia	Clostridiales	*Ruminococcaceae*	Unclassified	0.250 ± 0.547	0	0.047*
OTU_258	Firmicutes	Bacilli	Lactobacillales	*Streptococcaceae*	*Streptococcus*	0.0156 ± 0.0145	0.00448 ± 0.00570	0.049*

The OTUs of significant differences ($p<0.05$, Wilcoxon rank test) between CADASIL patients and controls (upper) and between the CADASIL patients with and without stroke (lower) are shown. *$p<0.05$, **$p<0.01$.
